# Contrasting Dynamics in Isoelectronic Anions Formed by Electron Attachment

**DOI:** 10.1021/acs.jpclett.3c03460

**Published:** 2024-01-19

**Authors:** P. Nag, M. Ranković, M. Polášek, R. Čurík, D. S. Slaughter, J. Fedor

**Affiliations:** † J. Heyrovský Institute of Physical Chemistry, The Czech Academy of Sciences, Dolejškova 3, 18223 Prague, Czech Republic; ‡ Chemical Sciences Division, Lawrence Berkeley National Laboratory, Berkeley, California 94720, United States

## Abstract

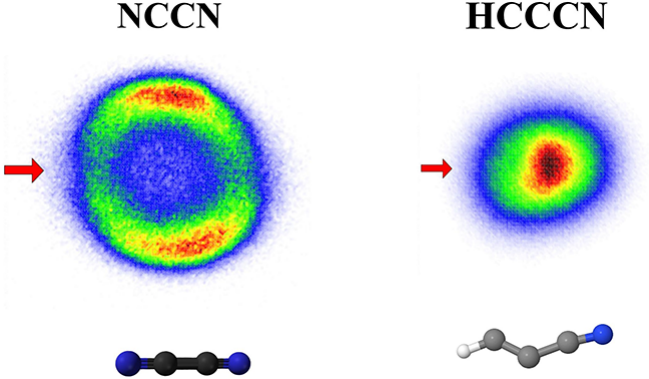

Cyanogen NCCN and cyanoacetylene HCCCN are isoelectronic molecules, and as such, they have many similar properties. We focus on the bond cleavage in these induced by the dissociative electron attachment. In both molecules, resonant electron attachment produces CN^–^ with very similar energy dependence. We investigate the very different dissociation dynamics, in each of the two molecules, revealed by velocity map imaging of this common fragment. Different dynamics are manifested both in the excess energy partitioning and in the angular distributions of fragments. Based on the comparison with electron energy loss spectra, which provide information about possible parent states of the resonances (both optically allowed and forbidden excited states of the neutral target), we ascribe the observed effect to the distortion of the nuclear frame during the formation of core-excited resonance in cyanoacetylene. The proposed mechanism also explains a puzzling difference in the magnitude of the CN^–^ cross section in the two molecules which has been so far unexplained.

Electron impact on molecules is a driving factor for chemical changes in many environments. It is often compared with processes initiated by a photon impact (e.g., photodissociation); however, there are significant differences between these two. One of the most important differences is the nature of the resonances in electron–molecule collisions. Electron–molecule resonances (temporary anions), like many electron–ion resonances, are electronic states embedded in the autoionization continuum. However, unlike photoionization shape resonances, for example, in temporary anion states, the outer valence electrons tend to be only weakly bound by electronic correlation in the potential of the neutral molecule. Dynamics on such states is often characterized by a strong coupling of electronic and nuclear motion, nonlocal, and nonadiabatic effects.^1^ A resonance has, in principle, two decay possibilities: (i) electron autodetachment and (ii) bond cleavage, resulting in anionic and neutral fragments (dissociative electron attachment, DEA). A useful experimental tool for probing the nuclear dynamics on resonances is to monitor these two decay channels and to use the fact that autodetachment can leave a molecule vibrationally excited.

In recent years, detailed insight into the nuclear dynamics on resonances has been obtained, especially by employing 2D vibrational electron energy loss spectroscopy.^2,3^ This approach, however, can be typically applied only to one-particle (shape) resonances. The two-particle resonances (electron is trapped by an excitation which it has induced) have typically much longer autodetachment lifetimes since the decay requires a change of the electronic configuration of the core.^4^ They thus rarely lead to vibrational excitation, and the nuclear dynamics has to be experimentally inferred solely on the basis of the DEA experiments. As in the whole field of molecular dynamics, important advances have been brought by anion fragment imaging,^5−7^ building upon the velocity map imaging (VMI) technique,^8^ with position- and time-sensitive detectors. In this Letter, we show how a combination of VMI with electron energy loss spectroscopy (EELS) in the electronic excitation range can explain a very different behavior of two isoelectronic linear molecules: cyanogen NCCN and cyanoacetylene HC_3_N.

The choice of these two molecules is not accidental. Among the simplest nitrogen-containing molecules, both have been identified as potential reactants or intermediates in the prebiotic synthesis of the building blocks of life.^9,10^ More specifically, the role of electron-induced dissociation is important in the chemistry of planetary and cometary atmospheres, and in interstellar clouds.^11^ Considering the low temperature and low particle density of interstellar matter, we observe a surprisingly high chemical diversity in the interstellar medium. Reactions leading to extraterrestrial synthesis are believed to be triggered externally, e.g., by X-rays, UV light, or electron impact. Cyanoacetylene and cyanogen are ubiquitous outside of the Earth. The former is one of the most abundant molecules in interstellar clouds. The direct spectroscopic detection of the latter is complicated by the lack of a dipole moment; however, detections of its protonated counterpart (NCCN)H^+^^12^ or of its polar isomer NCNC^13^ are strong hints that cyanogen is present in interstellar clouds in large quantities.^13^ Detected spectroscopically in outer space were also species related to cyanogen and cyanoacetylene such as radicals CN, C_3_N, and C_5_N^14^ or anions CN^–^ and C_3_N^–^.^15,16^ The astronomical relevance thus further motivates the additional interest in the electron-induced dynamics of these two molecules.

The DEA to both NCCN and HC_3_N leads to formation of CN^–^.^17−21^ The DEA cross sections,^19,21^ shown in Figure 1a,b, have very similar shapes, and both spectra have a dominant band peaking slightly above 5 eV. An important difference is that the cross section magnitude in NCCN is approximately by a factor of 6 larger than in HC_3_N. Figure 1c,d shows the velocity map images of CN^–^. In NCCN, the fragments have a higher kinetic energy release and a highly anisotropic angular distribution, with the preferential fragment emission being perpendicular to the electron beam. In contrast, the HC_3_N image shows an isotropic angular distribution with maximum intensity in the center, corresponding to low kinetic energy release. We ascribe the slight distortion of the image to the magnetic field present in the setup, as discussed in the Methods section. In addition to CN^–^, the DEA to HC_3_N leads also to C_3_N^–^, C_2_H^–^, and C_2_^–^ fragments. Their velocity map images are presented in the Supporting Information, and all have a character of a central blob, similar to that of CN^–^.

**Figure 1:**
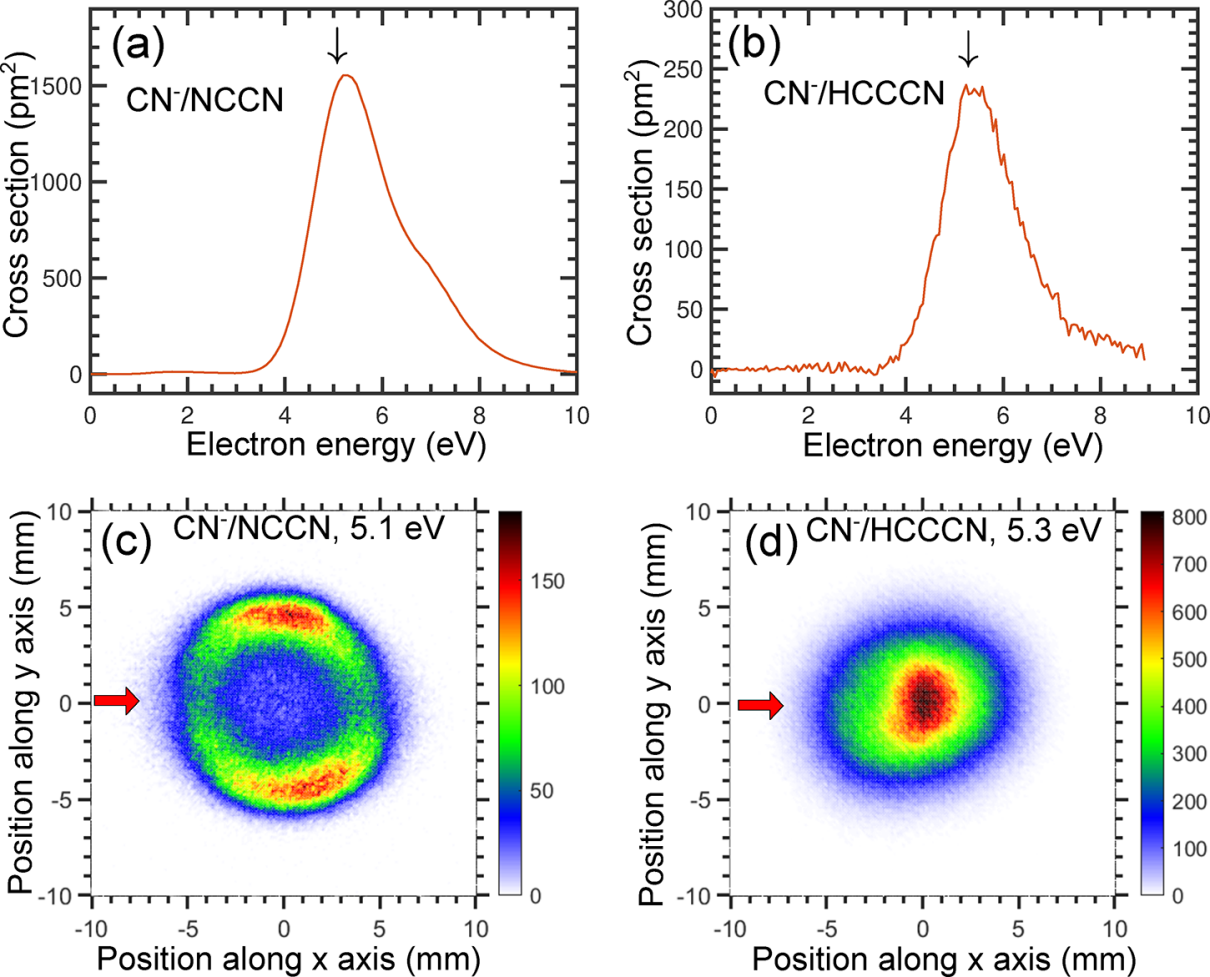
(a and b) DEA cross sections for the production of CN^–^ from NCCN and HC_3_N. (c and d) Central slices of velocity map images recorded at the electron energies denoted by vertical arrows in the top panels. The horizontal arrow in panel (c) denotes the direction of the electron beam.

Figure 2 shows the kinetic energy distributions (KEDs) obtained from the images. We have independently determined the KEDs using EELS, with the addition of a Wien filter set to detect anions instead of electrons, as described in the Methods section. The Wien filter discriminates between only electrons and anions without mass-resolving the latter. For NCCN, where the only fragment is CN^–^, the data from the two setups are thus directly comparable. For HC_3_N, the VMI KEDs of the four anionic fragments were weighted by their absolute cross sections, and this weighted sum is compared to the data from the EELS setup. The agreement between the KEDs from the two setups is excellent for HC_3_N and satisfactory for NCCN. We ascribe the observed difference mainly to the difference in resolution between the two setups (which is more than a factor of 10 worse in the VMI setup). Nonetheless, the VMI measurement is considered most reliable, as the measured yields are essentially independent of the anion KED, in contrast to the EELS measurement, which is subject to an anion energy-dependent analyzer response function that is expected to overestimate the anion yields at low energies. Still, the experimental conclusion is unambiguous: Compared to NCCN, DEA to HC_3_N produces much slower CN^–^ fragments which are emitted isotropically.

**Figure 2:**
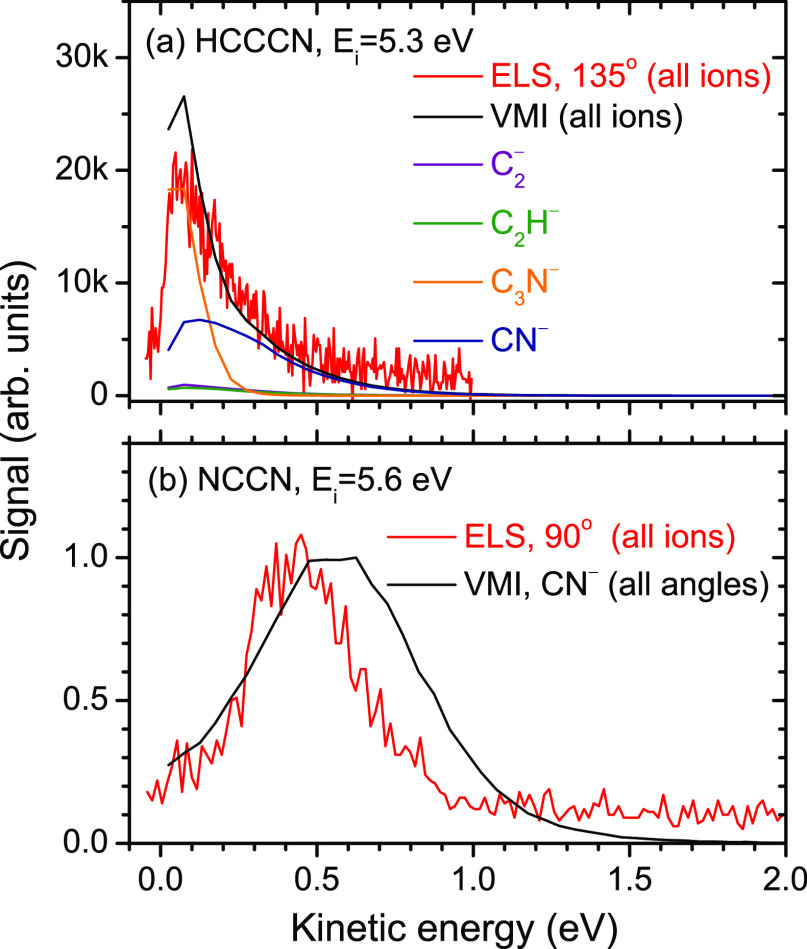
Kinetic energy distributions of anions produced in DEA to (a) HC_3_N and (b) NCCN. The black line is data from the VMIs, and the red line is the data from the EELS spectrometer (with the Wien filter in front of the detector set to record anions).

We first examine the reaction energetics. Table 1 lists the possibly relevant reaction channels. Among the pathways that produce excited fragments, we consider also the lowest excited state of CN^–^ (with a ^3^Σ term). This state lies energetically above the neutral and is thus not bound. Recently, Tian and co-workers^28^ suggested that DEA to BrCN produces long-lived excited CN^–^ and drew astrochemical implications from this fact. It is clear that in the cases of NCCN and HC_3_N, this fragment is energetically inaccessible at present incident energies. The only considerable difference between the two molecules is in the fact that the neutral counterparts in the dissociation, the CN and CCH radicals, have quite different energies of the lowest lying excited states (1.14 vs 0.46 eV). However, there is no reaction channel that could cause the very different CN^–^ KEDs in the two molecules purely from the different vertical and adiabatic reaction thresholds. Evidently, the topology of the anion resonance potential energy surface must play a decisive role in the dissociation dynamics.

**Table 1:** Energetics of Possible Reaction Channels^a^

Reaction	Anion	Neutral	*E*_*exc*_(CN^–^)	*E*_*exc*_(Neutral)	*E*_*th*_	*E*_*k*,*max*_(CN^–^)
e + NCCN	CN^–^(^1^Σ)	CN(^2^Σ)	0	0	2.11	1.5
	CN^–^(^1^Σ)	CN(^2^Π)	0	1.14^25^	3.25	0.93
	CN^–^(^3^Σ)	CN(^2^Σ)	≈3.5^b^	0	≈5.6	-
e + HC_3_N	CN^–^(^1^Σ)	CCH(^2^Σ)	0	0	2.51	1.37
	CN^–^(^1^Σ)	CCH(^2^Π)	0	0.46^25^	2.97	1.14
	CN^–^(^1^Σ)	CCH(^2^A^′^)	0	4.85^25^	7.35	-
	CN^–^(^3^Σ)	CCH(^2^Σ)	≈3.5^b^	0	≈6.0	-
	CN^–^(^1^Σ)	CC + H	0.	5.03^26^	7.74	-

a *E*_*exc*_(CN^–^) and *E*_*exc*_(Neutral) are the excitation energies of the fragments with respect to their ground states. *E*_*th*_ is the threshold energy. *E*_*k*,*max*_(CN^–^) is the maximal kinetic energy of CN^–^ if all the excess energy went into translational motion. It was evaluated for the incident energy of 5.1 eV for NCCN and 5.3 eV for HC_3_N. The data that were used for evaluating the thresholds: BDE(NC–CN) = 5.97 eV,^22^ BDE(NC–CCH) = 6.37 eV,^23^ EA(CN) = 3.86 eV.^24^.

b CN^–^(^3^Σ) is not a bound state and we are not aware of an experimental value for its energy. Calculations of its (resonant) energy have quite a large scatter depending on the used method: 3.5 eV,^27^ 4.2 eV,^28^ 6.5 eV.^29^ We tabulate the lowest of these values to show that this state is inaccessible at current incident energies.

In both NCCN and HC_3_N, a number of shape resonances have been identified experimentally (by electron transmission spectroscopy^30^ or by vibrational EELS^19,31^) and theoretically.^31−35^ In a simplified view, shape resonances are described by a temporal occupation of virtual molecular orbitals. Out of these, the σ_g_^*^ resonance in NCCN (ground state plus π_g_^*^ electron) and ^2^Π resonance in HC_3_N (ground state plus π* electron), both have centers around 5 eV and their profiles, as seen in the vibrational excitation cross sections, closely match the DEA bands. This fact has led in the past to a tentative assignment of this resonance to be responsible for the CN^–^ production.^17,20^ However, it has been noted (not only for these two molecules but also for a number of other polyines^36^) that these shape resonances might be too broad to yield such high DEA cross sections—the electron will autodetach faster than the nuclei dissociate.

The observed angular distribution of CN^–^/NCCN can help in identifying the resonant state. The influence of resonance symmetries on DEA angular distributions in diatomic molecules was developed by Taylor and O’Malley^37^ and later simplified and applied to small polyatomic molecules by Tronc et al.^38^ This theory assumes that (i) a single resonant state is involved, (ii) coupling is due to a pure electronic matrix element, and (iii) dissociation is so fast that the transient anion dissociation axis does not rotate (axial recoil approximation). The angular distribution then depends on the electronic state of the neutral target, electronic state of the resonance, and the partial wave of the incident electron. We applied this approach to NCCN under the assumption of a diatomic-like dissociation. The details of the model are provided in the Supporting Information. Figure 3 compares the experimental angular distribution with the distribution which would result from four possible resonant state symmetries: ^2^Σ_*g*_, ^2^Σ_*u*_, ^2^Π_*g*_, and ^2^Π_*u*_. The neutral target state in all of the cases is ^1^Σ_*g*_. The partial wave contributions for each resonant symmetry were obtained by a least-squares fitting to the experimental data. It is evident that the resonance symmetry most closely reproducing the experimental data is the Σ_*g*_. Each of the other symmetries have nodes at 0°, 90°, 180°, and/or 270°. The Σ_*g*_ resonance has an isotropic component originating from the *s*-wave contribution, with the maxima at perpendicular directions arising mainly from the *d*-wave. Even though this model has rather strict assumptions (diatomic-like dissociation, axial recoil approximation), it leads us to search for a resonance with Σ_*g*_ electronic symmetry.

**Figure 3:**
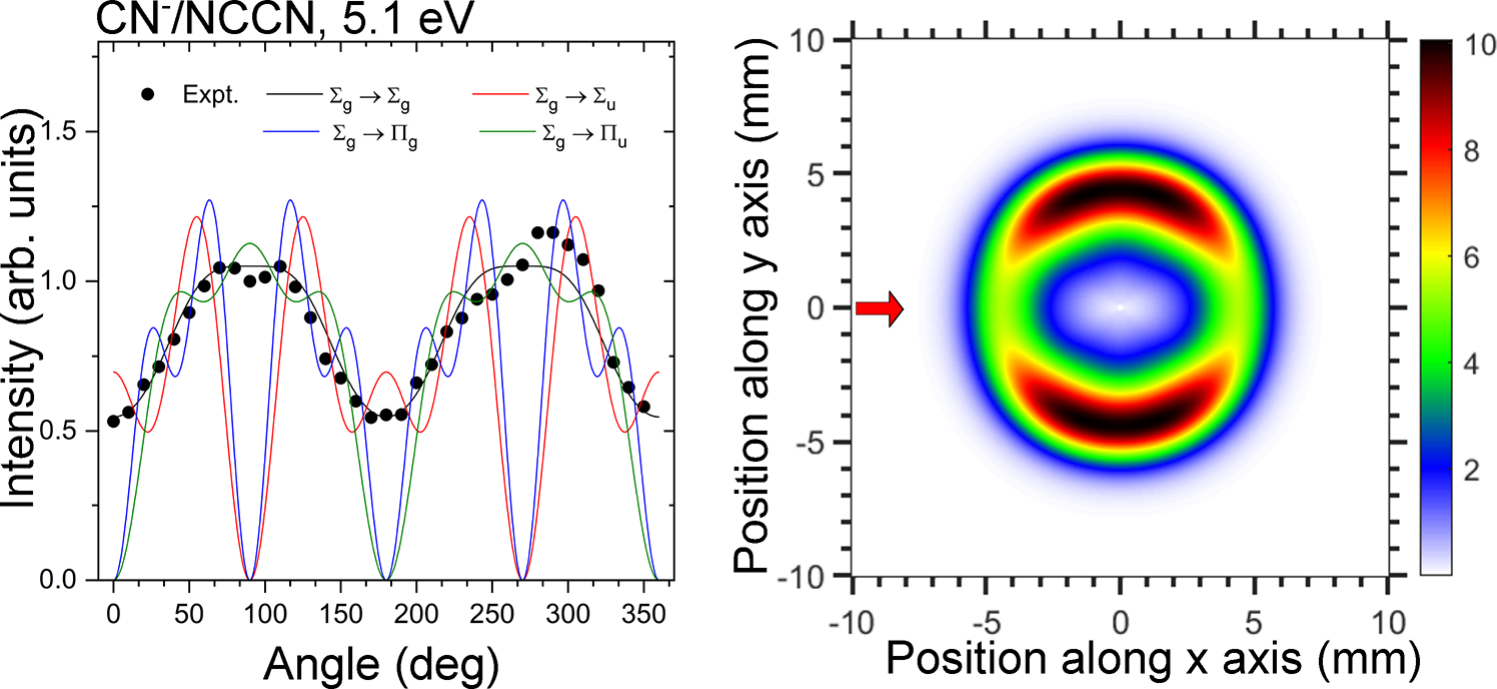
Left: Experimental angular distribution of CN^–^ from NCCN (points); fits of angular distributions were assumed to be based on various symmetries of resonant states. Right: Hypothetical CN^–^/NCCN image assuming a Σ_*g*_ resonant state and the experimentally measured KED.

Even though a shape resonance corresponding to temporal occupation of the σ_g_^*^orbital can be formed, it will be extremely short-lived due to a lack of barrier toward autodetachment. It would thus lead to negligible dissociation yields. For the relevant incident energies, DEA is most plausibly mediated by core-excited resonances.

The identification of core-excited resonances is facilitated by comparison with their parent states, i.e., electronically excited states of the neutral target molecules. These can be experimentally revealed by electron energy loss spectroscopy which carries the advantage of being able to reveal different types of states depending on the conditions at which the spectra are taken.^4,41^ At small scattering angles (near-forward direction) and at high energies, the dipole allowed transitions are almost exclusively observed and the energy loss spectrum strongly resembles the photoabsorption spectrum. A 1:1 correspondence between photoabsorption and EELS can be expected only for incident electron energies which are much higher than the energy loss;^42^ however, the energies of allowed transitions are revealed already at residual energies comparable to the energy loss.^41,43^ The optically forbidden transitions, including the electronic exchange interaction, are revealed at large scattering angles and low residual energies. Figure 4a shows the EELS of NCCN recorded at the scattering angle, θ = 10°, and residual energy *E*_*r*_ = 10 eV. All observed features are indeed in a perfect agreement with the VUV photoabsorption spectrum of Connors et al.^39^Figure 4b shows the EELS recorded at θ = 135° and *E*_*r*_ = 0.05 eV. Clearly, there is a group of states lying below 7 eV which are optically dark. The vertical bars denote the experimental onsets of transition as listed by Connors et al. (obtained from either their spectra or the earlier data). Figure 5 shows the same type of data for HC_3_N.

**Figure 4:**
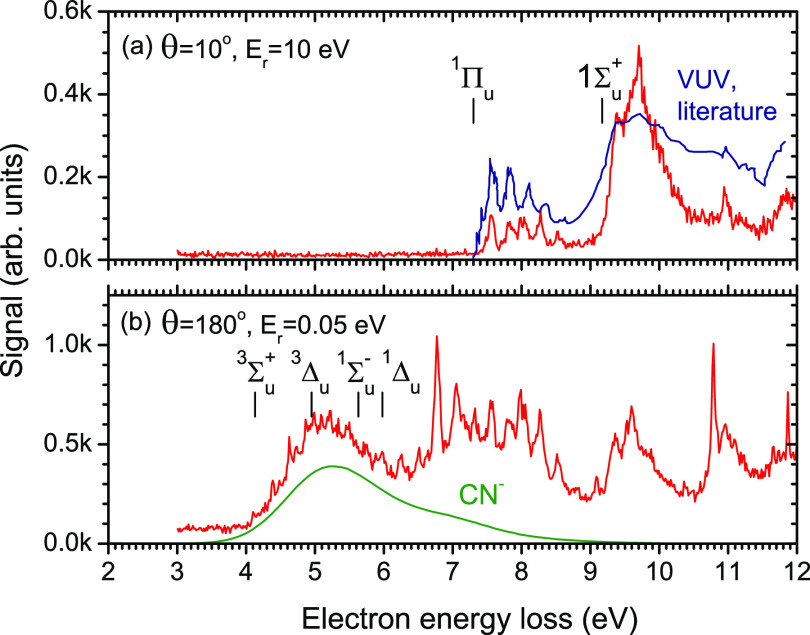
Red line: Electron energy loss spectra of NCCN recorded under two different scattering conditions. Blue line: VUV photoabsorption spectrum of NCCN from Connors et al.^39^ Green line: CN^–^/NCCN energy dependence arbitrarily scaled.

**Figure 5:**
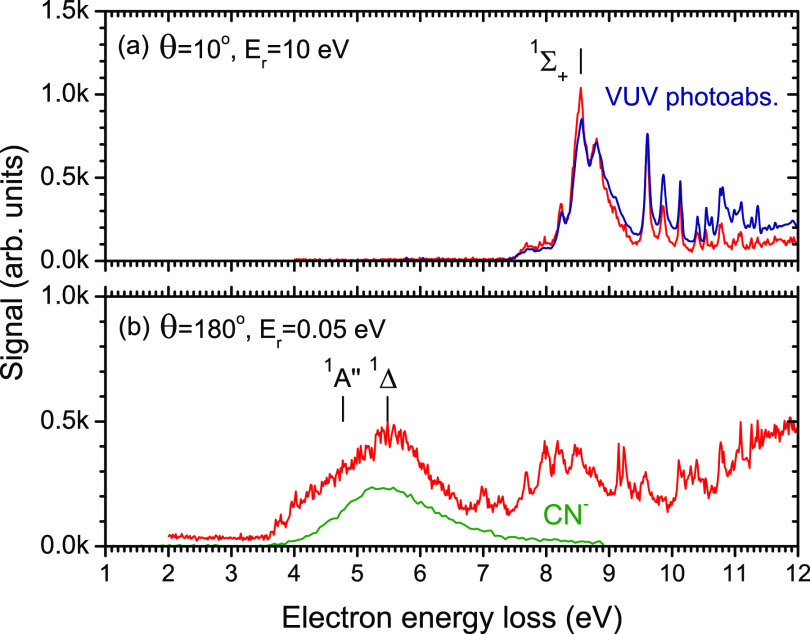
Red line: Electron energy loss spectra of HC_3_N recorded at two different scattering conditions. Blue line: VUV photoabsorption spectrum of HC_3_N from Ferradaz et al.^40^ Green line: CN^–^/HC_3_N energy dependence arbitrarily scaled.

The electronic configuration of neutral NCCN is [core]σ_*u*_^2^π_*u*_^4^σ_*g*_^2^π_*g*_^4^ with the unoccupied orbitals ordered as π_*u*_π_*g*_σ_*u*_.^44^ The configuration of all excited states marked in Figure 4 is π_*g*_^3^π_*u*_^1^.^44^ For the configurations that give rise to the Σ_*u*_ terms, if an extra electron will be added to the σ_*u*_ unoccupied orbital, the resulting core-excited resonance will have a Σ_*g*_ symmetry. Since such a resonant state would produce angular distribution in a perfect agreement with the experiment, we ascribe the CN^–^ production in NCCN to be mediated by a core-excited resonance with the configuration π_*g*_^3^π_*u*_^1^σ_*u*_^1^.

There is one important difference in the excited states of HC_3_N. It has been noted already by Job and King^45^ that the rotational structure in the electronic absorption spectra suggests that the lowest exited state is not linear, but has a trans-bent configuration. This has been confirmed later both experimentally^39^ and theoretically.^44,46^ The lowest excited state of HC_3_N marked in Figure 5 is ^1^A″. It is adiabatic, and the vertical excitation energies are 4.77 and 5.1 eV, respectively.^39,45^ The corresponding triplet state has been computationally predicted to be structurally similar to an adiabatic excitation energy of 3.43 eV.^46^ The present EELS spectrum in Figure 5b places its onset rather to 3.7 eV. These low-lying states (either singlet or triplet) are the most plausible candidates for the parent state of a core-excited resonance, which leads to CN^–^ production in HC_3_N.

We presume nonlinear geometry of the transient anion resonance is the reason for the slow CN^–^ fragments observed by the VMI measurement of Figure 1d. The incident electron simultaneously excites the HC_3_N molecule and gets temporarily trapped in its potential. The excitation is a vertical process; it thus proceeds while the nuclei are still in the linear geometry of the neutral. The initial momentum which the atomic nuclei get is thus in the bending direction. The bending is followed by an intramolecular vibration redistribution of the excess energy, which leads to a vibrationally hot transient anion state. The CN^–^ is then emitted isotropically with low kinetic energies. This is in strong contrast to NCCN, where, upon the resonance formation, the C–C bond is presumably directly dissociative, with the prompt dissociation producing fragments dissociating anisotropically and with high kinetic energy.

A crucial assumption of this hypothesis is that the HC_3_N^–^ resonant state, upon its vertical formation in the linear geometry, is unstable with respect to bending, while the NCCN^–^ state is not. In order to verify the plausibility of this assumption, we explored the excited states of both anions using the TD-DFT approach. Table 5 in the SI lists the excited states of NCCN^–^. The three lowest states with the ^2^Σ_*g*_ symmetry have the configurations 1^2^Σ_*g*_: π_*g*_^4^σ_*g*_^1^, 2^2^Σ_*g*_: σ_*g*_^1^π_*g*_^4^π_*u*_^2^, and 3^2^Σ_*g*_: π_*g*_^3^π_*u*_^1^σ_*u*_^1^. The first one would correspond to a very broad shape resonance; the second one would be a Feshbach-type resonance where the electron pair in the π_*u*_ orbital lowers the energy of the parent excited state, and the third one has the configuration hypothesized above from the comparison with EELS. Figure 6 shows that in contrast to the 2^2^Σ_*g*_ state, the 3^2^Σ_*g*_ state is directly dissociative along the CC bond. Also, the energy of NCCN^–^(3^2^Σ_*g*_) increases upon bending; its dissociation will thus proceed in the linear geometry. The equivalent state of HC_3_N^–^, 6^2^Σ_*g*_, is, however, unstable with respect to the bending of both the NCC and HCC angle.

**Figure 6:**
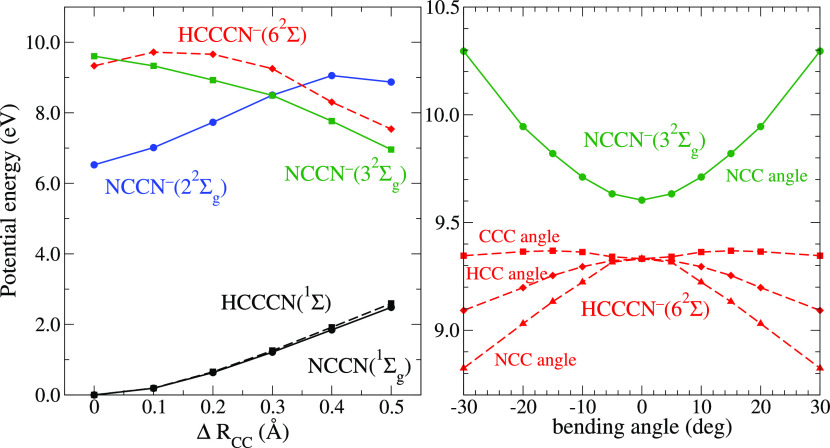
Left panel: Computed potential energy curves for the neutral ground state and selected Σ states of NCCN (full lines) and HCCCN (broken lines) molecules as functions of the C–C bond distance stretched by Δ*R*_*CC*_ from the equilibrium. Right panel: Potential energy curves of the ^2^Σ states of NCCN^–^ and HCCCN^–^ as functions of various bending angles. The energies shown in both panels are relative to the ground state energy of the corresponding neutral molecules at the equilibrium geometry.

A number of remarks should be added. First, due to the level of theory used, the energies of the calculated anion states in Figure 6 are expected to be higher than those of the corresponding resonant states, as explained in the Methods section. That is why they are consistently higher than the experimental positions of the DEA bands. Second, a useful comparison can be made with the photodissociation of HC_3_N. It proceeds via C–H bond cleavage which is the lowest neutral dissociation channel. Suits and co-workers^47^ reported that the dissociation on the S_1_ state (current ^1^A″) proceeds via an exit barrier of 0.2 eV. Figure 6 shows that a similar barrier appears in the C–C stretch coordinate of the resonant (6^2^Σ) surface, preventing its direct dissociation before bending. Our calculations indicate that this barrier is formed near a conical intersection with a higher state. This coupling of two Σ states is absent in the case of the NCCN molecules due to the higher (gerade and ungerade) symmetry. Third, the outlined mechanisms are perfectly consistent with the lower DEA cross section in HC_3_N observed experimentally. Fourth, apart from the geometry of the resonant state, another factor enhancing the intravibrational redistribution (IVR) in HC_3_N compared to NCCN is the higher number of internal degrees of freedom. This could facilitate a rapid coupling between the initial bending and other vibrational modes, distributing the resonance excess energy not into translational kinetic energy but into internal energy in one or both of the molecular fragments. Finally, since the dissociation competes with the electron autodetachment, it is important to note that the IVR does not necessarily need to be complete. The initial bending can lead to significant rotational excitation of the fragments, leading to slow CN^–^.

In conclusion, DEA to cyanogen and cyanoacetylene produces CN^–^ with very different dissociation dynamics. In NCCN, the VMI images and EELS spectra suggest that the core-excited resonance has a ^2^Σ_*g*_ (π_*g*_^3^π_*u*_^1^σ_*u*_^1^) configuration, which reproduces the data perfectly assuming a prompt dissociation of the resonance (prompt means that the axial recoil approximation is valid). In HC_3_N, the parent state of the equivalent core-excited resonance is nonlinear. We presume that after the vertical excitation, the bending dynamics leads to a fast IVR on the resonant potential energy surface (not necessarily complete) and consequently to an isotropic emission of CN^–^ with thermal energies. Our work provides the first evidence about how the electronic structure of the parent excited states influences the dynamics of the corresponding core-excited resonances.

## Methods

The DEA velocity map images have been measured on the DEA-VMI spectrometer.^21^ A magnetically cold-collimated pulsed electron beam (300 ns pulse width at 40 kHz) produced in a trochoidal electron monochromator crosses the effusive molecular beam of the target molecules. The produced anions are projected by an ion optics stack on a time- and position-sensitive delay-line detector. The fragment angular distribution is obtained from the central slice of a Newton sphere images, and the kinetic energy distributions are obtained from all ions in a 3D half-“Newton sphere”.^21^ The imaging spectrometer has mass resolution, which is enough to separate C_2_^–^, C_2_H^–^, and CN^–^ fragments of cyanoacetylene as shown in the SI. The absolute DEA cross sections have an uncertainly of ±15% for NCCN and ±20% for HC_3_N. The cross section calibration and the error budget are described in refs (19 and 21).

The electron energy loss spectra were measured on an electron spectrometer with hemispherical analyzers.^48,49^ The electron energy scale was calibrated on the ^2^S resonance in helium at 19.366 eV, and the electron-energy resolution was around 18 meV. A magnetic angle changer built around the collision region permits measurements in the full angular range, even at the normally inaccessible angles of 0° (forward scattering) and 180° (backward scattering). The EELS spectra (Figures 4 and 5) were measured in the mode with constant residual energy *E*_*r*_, where the potential of the analyzer is fixed and the potential of the monochromator is scanned to vary the incident energy *E*_*i*_. The signal is plotted as a function of energy loss Δ*E* = *E*_*i*_ – *E*_*r*_.

The analyzer is equipped with a Wien filter placed just before the channeltron and allows for selective detection of electrons or anions, albeit without resolving the individual ion masses. For the data shown in Figure 2, the Wien filter was set to record anions, the electron incident energy *E*_*i*_ was fixed, and the *E*_*r*_ (which here corresponds to ion kinetic energy) was scanned.

The cyanogen sample was prepared by a modified literature procedure^50^ in which an aqueous solution of potassium cyanide (11.6 g in 50 mL of water) was poured onto 22.3 g of copper(II) sulfate pentahydrate powder at a temperature rising from 50 to 90 °C. Evolving NCCN was passed through a Dimroth condenser to remove the water vapor. Then it was captured in a flask cooled down by a mixture of dry ice and acetone to a temperature of −78 °C. The cyanoacetylene was synthesized by the dehydration of the propiolamide, prepared by the reaction of methylpropiolate and ammonia, the method introduced by Miller and Lemmon.^51^

All the potential energy surfaces presented here have been calculated by the time-dependent DFT (TD-DFT) method employing the B3LYP functional as implemented in Gaussian 16.^52^ The TD-DFT calculations used the cc-pVTZ basis set,^53^ and they were applied to the neutral singlet and triplet excited states, as well as anions’ excited doublets. While this method appeared to be very stable with respect to the decay of the anion states into diffused continua (describing the neutral and an additional electron in the most diffused orbital), it is important to also mention the limitations of these calculations:

- The compact valence triple-ζ basis^53^ stabilized the anion states but it also likely causes an overestimate of the corresponding resonant energies. However, we believe that the qualitative behavior of the resonance surface upon bending and stretching of the molecular bonds should be correct with the present method.
- Not every computed negative ion state corresponds to a physical resonance as there is no computed information about lifetimes of these states
- Singlet calculations employ restricted determinants of the B3LYP functional, while the triplet and doublet calculations are unrestricted. Unfortunately, the degenerate Π, Δ, Φ, ... states are split energetically in the unrestricted calculations and they must be identified as pairs by an inspection of their orbitals.

## Supporting Information Available

(i) Experimental data on DEA to HC_3_N (mass spetrum, VMI images, and KEDs for all anionic fragments), (ii) symmetry analysis of CN^–^ angular distribution in NCCN, and (iii) configurations and calculated energies of excited neutral and anion states (PDF)
