# Supplementary material for: Contrasting Dynamics in Isoelectronic Anions Formed by Electron Attachment
Source: J Phys Chem Lett. 2024 Jan 19;15(4):895–902. doi: 10.1021/acs.jpclett.3c03460 (PMC10839900; doi:10.1021/acs.jpclett.3c03460)
Supplement: Supplementary file 1 — jz3c03460_si_001.pdf [file jz3c03460_si_001.pdf]

SUPPLEMENTARY INFORMATION for Contrasting Dynamics in Isoelectronic Anions Formed by Electron Attachment

P. Nag,[†] M. Ranković,[†] M. Polášek,[†] R. Čurík,[†] D. S. Slaughter,[‡] and J. Fedor[*,†]

†*J. Heyrovský Institute of Physical Chemistry, The Czech Academy of Sciences, Dolejškova 3, 18223 Prague, Czech Republic*

‡*Chemical Sciences Division, Lawrence Berkeley National Laboratory, Berkeley, CA 94720, USA*

E-mail: juraj.fedor@jh-inst.cas.cz

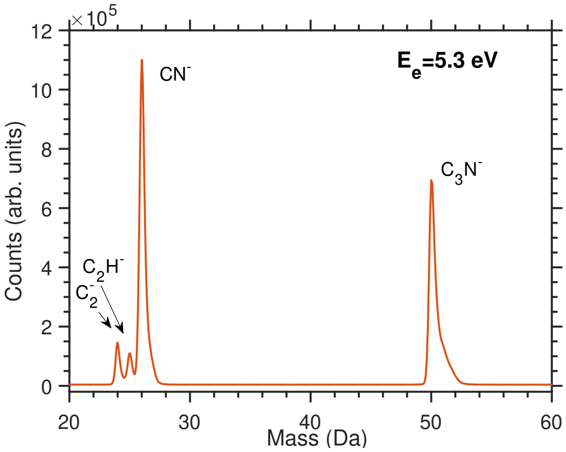

Figure 1: Mass spectrum of DEA fragments from HC_3N at 5.3 eV electron energy.

DEA to HC_3N

Here we present additional experimental data on the DEA to cyanoacetylene. Figure 1 shows the time-of-flight mass spectrum of anionic fragments which demonstrates that the individual ions are resolved and that the central slice images do not contain significant contribution of ions with neighbouring masses.

Figure 2 shows the images of the four fragments recorded at 5.3 eV incident electron energy. The general character of the image (central blob which is typical for a statistical distribution of kinetic energies) is the same for all the fragments and does not change at different incident energies. Figure 3 shows the kinetic energy distributions of fragments obtained from the images. The distributions change only very weakly with the electron energy.

DEA to NCCN: analysis of angular distributions

A theory relating the angular distribution of the anionic fragments produced due to dissociative electron attachment (DEA) and the symmetry of the resonance state was given by O'Malley and Taylor.[1] The theory was developed for diatomic molecules and considering only a single resonance state is involved. The coupling is due to pure electronic matrix

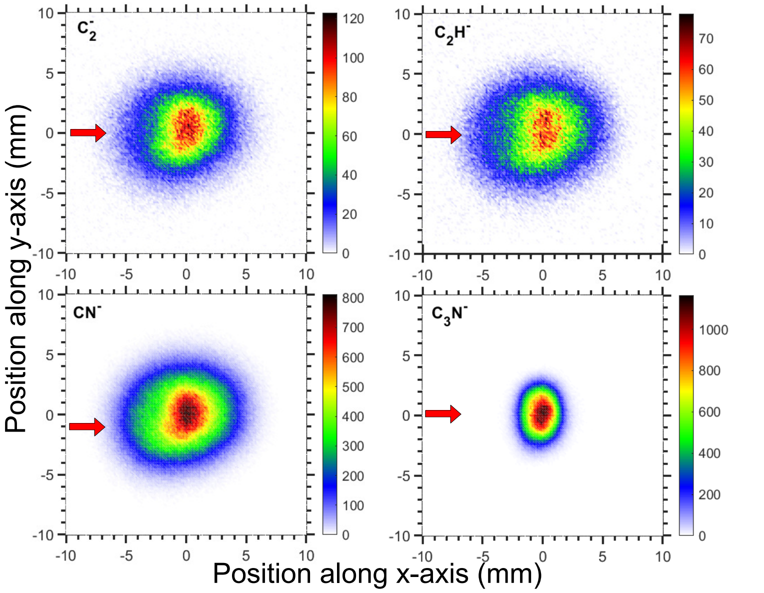

Figure 2: VMI images of four anionic fragments produced due to DEA to HC_3N molecule at 5.3 eV incident electron energy. Each images are of 5 ns width around the centre of the 'Newton sphere' and the small red arrows indicate the direction of the incident electron beam.

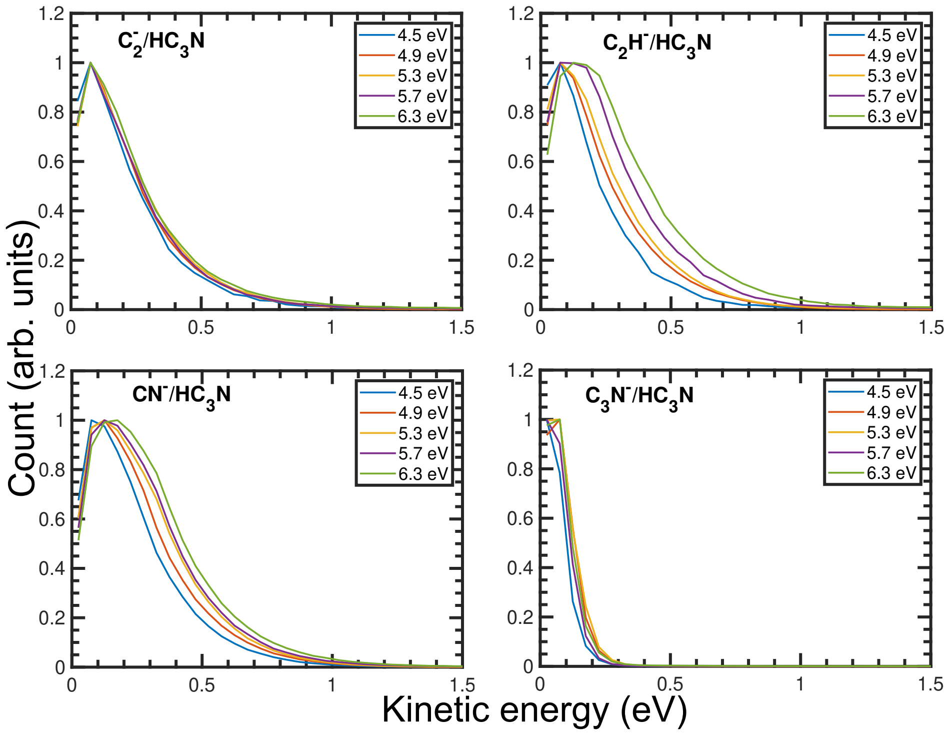

Figure 3: Kinetic energy distribution of all the four anionic fragments produced due to DEA to HC_3N molecule for different incident electron energies as indicated are shown here.

elements (independent of spin) considering axial recoil approximation is valid i.e., during the dissociation rotation of the ions is negligible. With the same assumptions Azria *et al.*[2] later generalised this theory to treat polyatomic molecules. The angular distribution of the anionic fragment in the laboratory frame with respect to the incident electron beam direction ϑ can be expressed as $I^\epsilon(\vartheta)$ for each resonant state ϵ, integrating of the azimuthal angle φ

$$I^\epsilon(\vartheta) \propto \frac{1}{2\pi} \int_0^{2\pi} \left| \sum_{l,m} a_{l,m} i^l e^{i\delta_l} X^\epsilon_{l,m}(\vartheta, \varphi) \right|^2 d\varphi, \tag{1}$$

where ϑ and φ are the polar angles of the electron beam, $X^\epsilon_{l,m}(\vartheta, \varphi)$ are the basis functions for the irreducible representations of the point group G of the molecule in the dissociation frame. The expansion coefficients $a_{l,m}$ are real numbers, whereas the δ_l are the phase differences between partial waves with respect to the lowest one involved.

Based on equation (1) the expression in equation (2) was obtained and used to fit the experimentally obtained angular distribution data.

$$I(\vartheta) \sim \left| \sum_{l=|\mu|} a_l Y_{l,\mu} e^{i\delta_l} \right|^2. \tag{2}$$

In the above expression, the quantity μ corresponds to the change in orbital angular momentum between the initial and the final molecular state. It can be defined as, $\mu = |\Lambda_f - \Lambda_i|$, where Λ_i and Λ_f are the projection of the electronic axial orbital momentum along the molecular axis for the initial and final molecular states, respectively. The summation index $l \geq |\mu|$ can further be restricted between only even or odd values depending on whether the initial and final state have the same parity or not. The a_l's are the expansion coefficients of each partial wave of the spherical harmonics $Y_{l,\mu}$.

To compute the hypothetical CN^-/NCCN velocity map image (shown in Fig. 3 of the main text), the involvement of a Σ_g resonant state is considered. As both the initial and final states are of Σ_g symmetries, the change in orbital angular momentum, $\mu = 0$ and also due to the same parity of both states, the values of l are further restricted only to even

numbers. In the present case, the first three partial waves with l=0, 2 and 4 which can lead a Σ_g to Σ_g transition are considered. The values of different fitting parameters obtained for the best fit of the angular distribution which was further used to compute the hypothetical VMI image are as follows: a_0 =3.306; a_2 =0.5901; a_4 =0.1399; d_2 =4.159; d_4 =3.657. As already defined, d_2 and d_4 are the phase differences between the s-wave (with $l = 0$) and d-wave (with $l = 2$) and in-between s-wave and g-wave (with $l = 4$), respectively and a_l's are the expansion coefficient of each partial waves, with the given l values.

Time-dependent DFT study of NCCN and HCCCN

The TD-DFT calculations employed the B3LYP functional and the cc-pVTZ basis set[3] as implemented in Gaussian 16.[4] The neutral and anion excited states will be referred with respect to the following ground state configurations:

$$\begin{aligned} \text{NCCN} &: (1\sigma_u)^2(1\sigma_g)^2(2\sigma_g)^2(2\sigma_u)^2(3\sigma_g)^2(3\sigma_u)^2(4\sigma_g)^2(1\pi_u)^4(4\sigma_u)^2(5\sigma_g)^2(1\pi_g)^4 \\ \text{HCCCN} &: (1\sigma)^2\cdots(8\sigma)^2(1\pi)^4(9\sigma)^2(2\pi)^4 \end{aligned}$$

Excited neutral states

Table 1: Vertical energies of the excited singlet states of NCCN.

State	Energy (eV)	Symmetry	Note
1	5.77	Σ_u	$(1\pi_g)^3(2\pi_u)^1$
2	5.99	Δ_u	$(1\pi_g)^3(2\pi_u)^1$
3	5.99	Δ_u	$(1\pi_g)^3(2\pi_u)^1$
4	7.66	Π_u	$(5\sigma_g)^1(2\pi_u)^1$
5	7.66	Π_u	$(5\sigma_g)^1(2\pi_u)^1$
6	7.93	Σ_g	$(1\pi_u)^3(2\pi_u)^1$
7	8.09	Π_g	$(4\sigma_u)^1(2\pi_u)^1$
8	8.09	Π_g	$(4\sigma_u)^1(2\pi_u)^1$

Vertical energies of the triplets are calculated as excitations of the tripled ground state. For the electron-molecule collision purposes it is convenient to present them relative to the

Table 2: Vertical energies of the excited triplet states of NCCN relative to the singlet ground state.

State	Energy (eV)	Symmetry	Note
1	4.95	Σ_u	$(1\pi_g)^3(2\pi_u)^1$
2	5.50	Δ_u	$(1\pi_g)^3(2\pi_u)^1$
3	5.50	Δ_u	$(1\pi_g)^3(2\pi_u)^1$
4	6.62	Π_u	$(5\sigma_g)^1(2\pi_u)^1$
5	6.94	Σ_g	$(4\sigma_u)^1(2\pi_u)^1$

Table 3: Vertical energies of the excited singlet states of HCCCN.

State	Energy (eV)	Symmetry	Note
1	5.25	Σ	$(2\pi)^3(3\pi)^1$
2	5.42	Δ	$(2\pi)^3(3\pi)^1$
3	5.42	Δ	$(2\pi)^3(3\pi)^1$
4	7.67	Σ	$(1\pi)^3(3\pi)^1$
5	7.85	Π	$(9\sigma)^1(3\pi)^1$
6	7.85	Π	$(9\sigma)^1(3\pi)^1$
7	9.02	Δ	$(1\pi)^3(3\pi)^1$
8	9.02	Δ	$(1\pi)^3(3\pi)^1$

Table 4: Vertical energies of the excited triplet states of HCCCN relative to the singlet ground state.

State	Energy (eV)	Symmetry	Note
1	4.49	Σ	$(2\pi)^3(3\pi)^1$
2	4.99	Δ	$(2\pi)^3(3\pi)^1$
3	4.99	Δ	$(2\pi)^3(3\pi)^1$
4	6.62	Π	$(9\sigma)^1(3\pi)^1$
5	7.12	Δ	$(1\pi)^3(3\pi)^1$
6	7.51	Δ	$(1\pi)^3(3\pi)^1$

singlet neutral ground state as shown in Tables 2 and 4. Note that all the energies listed here are relative to the neutral ground state in the equilibrium geometry. Therefore, their values will be generally higher when compared to the calculations of Fischer and Ross[5] that list the adiabatic (geometry-relaxed) energies of the excited states.

Negative ion states of NCCN

Energies of the negative ion states presented here are relative to the neutral ground state in the equilibrium geometry. Values are summarized in Tab. 5.

Table 5: Vertical energies of the doublet negative ion states of NCCN relative to the neutral ground state. Bolded are the three $^2\Sigma_g$ states relevant for the article discussion.

State	Energy (eV)	Symmetry	Configuration, resonance type, note
1	-0.04	$1^2\Pi_u$	$(1\pi_g)^4(2\pi_u)^1$, shape, reported at +0.22 eV in Nag et al.[6]
2	-0.04	$1^2\Pi_u$	$(1\pi_g)^4(2\pi_u)^1$, shape, reported at +0.22 eV in Nag et al.[6]
**3**	**4.11**	$\mathbf{1^2\Sigma_g}$	$(1\pi_g)^4(6\sigma_g)^1$, shape, not seen in Nag et al.[6]
4	4.56	$1^2\Sigma_u$	$(1\pi_g)^4(5\sigma_u)^1$, shape, reported at 4.3 eV in Nag et al.[6]
5	4.59	$1^2\Pi_g$	$(1\pi_g)^3(2\pi_u)^2$, Feshbach, parents 1–3 in Tabs. 1 and 2
6	4.99	$1^2\Pi_g$	$(1\pi_g)^3(2\pi_u)^2$, Feshbach, parents 1–3 in Tabs. 1 and 2
7	5.44	$2^2\Pi_g$	$(1\pi_g)^3(2\pi_u)^2$ + $(1\pi_g)^4(2\pi_g)^1$, Feshbach + shape
8	5.66	$2^2\Pi_g$	$(1\pi_g)^3(2\pi_u)^2$ + $(1\pi_g)^4(2\pi_g)^1$, Feshbach + shape
9	5.85	$3^2\Pi_g$	$(1\pi_g)^3(2\pi_u)^2$ + $(1\pi_g)^4(2\pi_g)^1$, Feshbach + shape
10	6.15	$3^2\Pi_g$	$(1\pi_g)^3(2\pi_u)^2$ + $(1\pi_g)^4(2\pi_g)^1$, Feshbach + shape
**11**	**6.53**	$\mathbf{2^2\Sigma_g}$	$(5\sigma)^1(2\pi_u)^2$, Feshbach, parents 4,5 in Tab. 1 or 4 in Tab. 2
12	6.71	$2^2\Pi_u$	$(1\pi_u)^3(2\pi_u)^2$, Feshbach, parent 6 in Tab. 1
13	6.84	$2^2\Sigma_u$	$(4\sigma_u)^1(2\pi_u)^2$, Feshbach, parent 7 in Tab. 1
14	7.17	$2^2\Pi_u$	$(1\pi_u)^3(2\pi_u)^2$, Feshbach, parent 6 in Tab. 1
⋮	⋮	⋮	⋮
**29**	**9.60**	$\mathbf{3^2\Sigma_g}$	$(1\pi_g)^3(2\pi_u)^2(5\sigma_u)^1$, core-excited

References

(1) O'Malley, T. F.; Taylor, H. S. Angular Dependence of Scattering Products in Electron-Molecule Resonant Excitation and in Dissociative Attachment. *Phys. Rev.* **1968**, *176*, 207–221.

(2) Azria, R.; Coat, Y. L.; Lefevre, G.; Simon, D. Dissociative electron attachment on H2S: energy and angular distributions of H- ions. *J. Phys. B* **1979**, *12*, 679–687.

(3) Dunning, T. H. Gaussian Basis Sets for Use in Correlated Molecular Calculations. I. The Atoms Boron Through Neon and Hydrogen. *J. Chem. Phys.* **1989**, *90*, 1007–1023.

(4) Frisch, M. J.; Trucks, G. W.; Schlegel, H. B.; Scuseria, G. E.; Robb, M. A.; Cheeseman, J. R.; Scalmani, G.; Barone, V.; Petersson, G. A.; Nakatsuji, H. et al. Gaussian˜16 Revision C.01. 2016; Gaussian Inc. Wallingford CT.

(5) Fischer, G.; Ross, I. G. Electronic Spectrum of Dicyanoacetylene. 1. Calculations of the Geometries and Vibrations of Ground and Excited States of Diacetylene, Cyanoacetylene, Cyanogen, Triacetylene, Cyanodiacetylene, and Dicyanoacetylene. *J. Phys. Chem. A* **2003**, *107*, 10631–10636.

(6) Nag, P.; Čurík, R.; Tarana, M.; Polášek, M.; Ehara, M.; Sommerfeld, T.; Fedor, J. Resonant States in Cyanogen NCCN. *Phys. Chem. Chem. Phys.* **2020**, *22*, 23141.
